# Iodine Nutritional Status and Its Associated Factors Among Children and Adolescents in Zhejiang Province Ten Years After the Downward Adjustment of the National Salt Iodization Policy

**DOI:** 10.3390/nu18101634

**Published:** 2026-05-21

**Authors:** Ziying Jiang, Simeng Gu, Hui Kan, Yan Zou, Lichun Huang, Fanjia Guo, Sujun Yan, Yuanyang Wang, Zhijian Chen, Xiaofeng Wang, Xiaoming Lou, Guangming Mao, Zhe Mo

**Affiliations:** 1 Zhejiang Provincial Center for Disease Control and Prevention, Hangzhou 310051, China; m13736587731@163.com (Z.J.); smgu@cdc.zj.cn (S.G.); kanhui@zcmu.edu.cn (H.K.); yzou@cdc.zj.cn (Y.Z.); lchuang@cdc.zj.cn (L.H.); fjguo@cdc.zj.cn (F.G.); sjyan@cdc.zj.cn (S.Y.); yywang@cdc.zj.cn (Y.W.); zhjchen@cdc.zj.cn (Z.C.); xfwang@cdc.zj.cn (X.W.); xmlou@cdc.zj.cn (X.L.); 2 School of Public Health, Zhejiang Chinese Medical University, No. 548 Binwen Rd., Hangzhou 310053, China

**Keywords:** children and adolescents, urinary iodine, iodine nutritional status, iodized salt, cross-sectional study

## Abstract

Background: Iodine nutrition requires continued surveillance after changes in salt iodization policy. This study evaluated iodine status and associated factors among children and adolescents in Zhejiang Province, ten years after the national salt iodization standard was lowered. Methods: A cross-sectional survey employing a stratified, multistage cluster sampling design was conducted in 2022. A total of 688 participants aged 6–17 years with complete data on urinary iodine concentration, household salt iodine concentration, geographic classification, and key questionnaire variables were included in the analysis. Multivariate logistic regression analysis was performed to identify factors independently associated with iodine sufficiency. Results: Among 688 participants, the median household salt iodine concentration was 21.50 mg/kg, and iodized salt coverage was 64.68%. The median urinary iodine concentration (UIC) was 191.4 μg/L; however, 15.26% of participants had UIC < 100 μg/L. Participants in coastal areas had lower UIC levels and lower household iodized salt coverage than those in inland areas. Multivariate logistic regression analysis identified age, geographic region, and household use of iodized salt as factors significantly associated with iodine sufficiency. Conclusions: The overall iodine nutritional status among children and adolescents aged 6–17 years in Zhejiang Province is adequate. However, a certain proportion of iodine deficiency persists. Continued, targeted monitoring and health education on the appropriate use of qualified iodized salt are warranted, particularly in coastal areas and among younger children.

## 1. Introduction

Iodine is an essential micronutrient for the human body, playing a critical role in growth and development, energy metabolism regulation, and maintenance of cognitive function in children and adolescents [1,2]. Children and adolescents are in a stage of rapid growth and development and are more sensitive to changes in iodine nutritional status. Insufficient iodine intake can be associated with goiter, thyroid dysfunction, and varying degrees of growth, developmental, and intellectual impairment. Conversely, excessive iodine intake may also increase the risk of structural and functional thyroid abnormalities, including hyperthyroidism and autoimmune thyroid diseases [3,4]. Therefore, maintaining an appropriate iodine nutritional level remains an important issue in the fields of child and adolescent nutrition and public health.

Following the implementation of the universal salt iodization strategy, the overall iodine nutritional status of the Chinese population has significantly improved, and remarkable achievements have been made in the prevention and control of iodine deficiency disorders [5,6]. However, disparities in iodine nutritional status may still exist across different regions and groups. The median urinary iodine concentration (UIC) is a commonly used indicator for assessing the recent iodine intake and nutritional status of a population, and it serves as an important basis for population iodine nutrition surveillance [7]. Previous studies have indicated that the iodine nutritional status of children and adolescents is not influenced by a single factor but is associated with multiple factors, including regional environment, household iodized salt consumption, dietary behaviors, and individual characteristics [8].

Worldwide, although universal salt iodization has significantly improved population iodine nutrition, iodine deficiency remains a public health concern in certain regions and specific populations [9,10]. School-aged children and adolescents are key populations for iodine nutrition monitoring, as their urinary iodine levels can reflect recent iodine intake in response to dietary intake and are commonly used for population-level iodine nutrition assessment [10]. Variations in iodine nutrition status among children across different countries and regions may be attributed to differences in dietary patterns, coverage of iodized salt, and public health policies [11,12]. Notably, the intake of seafood exhibits substantial individual variability, making it an unreliable and stable alternative to iodized salt as a source of iodine [10,13]. Since the implementation of the universal salt iodization policy in 1994, significant achievements have been made in the prevention and control of iodine deficiency disorders in Zhejiang Province [14,15]. However, with the improvement of residents’ living standards and health awareness, changes in dietary structure and salt consumption behavior, and particularly after the adjustment of the Edible Salt Iodine Content standard in 2012 [16,17], the consumption rate of qualified iodized salt in households in Zhejiang Province has shown a declining trend. This may further be associated with the iodine intake levels and iodine nutritional status of children and adolescents [18,19,20,21]. Against this background, reassessing the iodine nutritional status of children and adolescents a decade after the national salt iodization policy adjustment holds profound practical significance and public health value. Zhejiang Province encompasses coastal, near-coastal, and inland areas, where residents may exhibit differences in salt choices and dietary patterns. Therefore, the regional heterogeneity in iodine nutritional levels among children and adolescents and its associated factors warrant further attention.

Although previous studies have described the iodine nutritional status of residents in certain regions of China, research focusing on children and adolescents as a key population remains relatively limited. In particular, there is a paucity of studies covering a broad age range while simultaneously integrating household salt iodine content, individual urinary iodine levels, and regional disparities. Previous research conducted in Zhejiang Province has predominantly focused on children aged 8–10 years [19] or has been based largely on routine surveillance data, which is insufficient to comprehensively reflect the iodine nutritional status and regional heterogeneity of children and adolescents aged 6–17 years, a decade after the downregulation of the salt iodization standard. Compared with previous studies, the present investigation has the following characteristics: First, the study population encompasses children and adolescents aged 6–17 years, thus covering a broader age range. Second, it concurrently incorporates individual urinary iodine concentration and household salt iodine content, enabling a comprehensive assessment of iodine nutritional status at both the individual and household levels. Third, it further compares differences among coastal, near-coastal, and inland areas to identify regions and groups that may require prioritized attention. Accordingly, this study utilized data from the 2022 Zhejiang Province Nutrition and Health Survey to describe the iodine nutritional status of children and adolescents aged 6–17 years, to compare differences in urinary iodine levels among groups stratified by age, sex, region, and lifestyle, and to analyze the associations between factors such as age, region, and household use of iodized salt and iodine nutritional status. The findings of this study may provide a basis for iodine nutritional surveillance, health education, and targeted interventions for children and adolescents in Zhejiang Province and similar coastal regions.

## 2. Materials and Methods

### 2.1. Sampling and Participants

A cross-sectional survey was conducted in 2022 using a representative sample of children and adolescents in Zhejiang Province. A stratified multi-stage sampling technique was employed. First, 16 cities/districts in Zhejiang Province were randomly selected as monitoring sites. Second, three towns or streets were randomly selected from each chosen site. Third, two villages or residential committees were randomly selected from each chosen town or street.

Inclusion criteria were as follows: residence in the surveyed county for 3 years or more; good past health with no history of thyroid diseases such as hyperthyroidism, hypothyroidism, or autoimmune thyroiditis. Exclusion criteria included the removal of participants with missing data on indicators such as urinary iodine, household salt iodine content, or geographic region.

**Figure 1:**
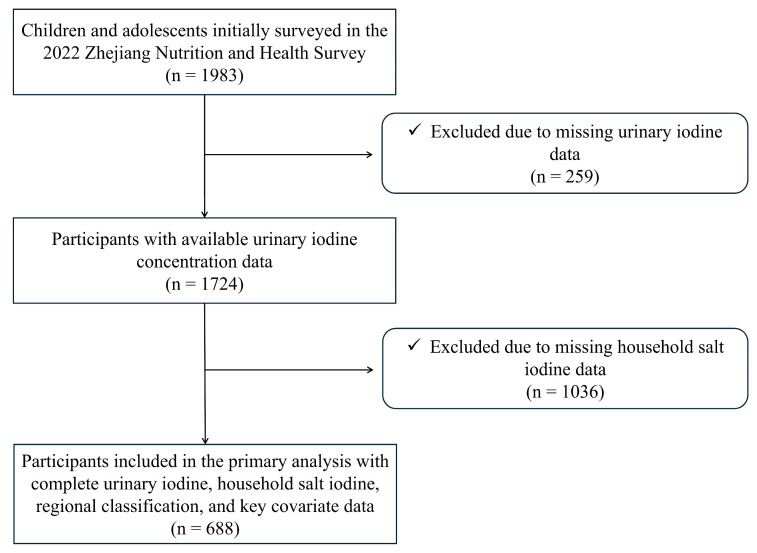
Flow diagram of participant inclusion and exclusion.

A total of 1983 children and adolescents aged 6 to 17 years participated in the 2022 Zhejiang Province Nutrition and Health Survey. Among them, 259 participants were excluded due to the absence of urinary iodine concentration data, resulting in 1724 participants with available urinary iodine data. Given that the present study aimed not only to characterize urinary iodine status but also to investigate the associations between iodine nutritional status, household salt iodine content, and regional disparities, participants lacking complete household salt iodine data and corresponding geographic classification information were further excluded. Ultimately, 688 participants with complete data on urinary iodine concentration, household salt iodine content, geographic classification, and key questionnaire responses were included in the primary analysis. Participants were stratified into two age groups: 6–11 years and 12–17 years. The participant selection process is illustrated in Figure 1.

### 2.2. Data and Sample Collection

The study primarily consisted of two components: a questionnaire covering basic information, personal health, and behavioral factors, and the detection of salt iodine and urinary iodine. The questionnaire included: (1) Basic information: age, sex, region, height, weight, and household iodized salt use. (2) Personal health and behavioral factors: dietary characteristics, eating habits, taste preferences, frequency of eating out, sedentary behavior, sleep disorders, sleep insufficiency, and moderate-to-vigorous physical activity (MVPA). All variables were collected using the standardized questionnaire of the 2022 Zhejiang Province Nutrition and Health Survey. Detailed definitions and classification criteria are presented in Supplementary Table S1.

For iodine-related testing, a salt sample of no less than 30 g was collected from each participant’s household. The sample was sealed, stored away from sunlight, and prepared for analysis. Additionally, a single casual urine sample of no less than 5 mL was collected from each participant. The urine sample was sealed, transported, and stored at −20 °C until analysis. After collection, salt and urine samples were stored and transported separately.

### 2.3. Measurements and Determination Criteria

#### 2.3.1. Iodized Salt

The iodine content in household edible salt was determined by sodium thiosulfate titration according to the General Test Method for Salt Industry—Determination of Iodine (GB/T 13025.7-2012) [22]. The brief procedure is as follows: A quantitative salt sample was weighed and dissolved in distilled water. Iodine was liberated under acidic conditions. Using starch as an indicator, the liberated iodine was titrated with a standardized sodium thiosulfate solution. The iodine concentration was calculated based on the titration volume and sample mass, and the results were expressed in mg/kg. According to the Iodine Content in Edible Salt (GB 26878-2011) [17], the iodine fortification level for edible salt in Zhejiang Province is 25 mg/kg, with an allowable fluctuation range of 18–33 mg/kg. Salt with an iodine content of <5 mg/kg was defined as non-iodized salt. Salt with an iodine content of 5–<18 mg/kg or >33 mg/kg was defined as non-compliant iodized salt. Salt with an iodine content of 18–33 mg/kg was defined as qualified iodized salt.

The coverage rate of iodized salt was calculated as the proportion of households using iodized salt among all surveyed households, using the formula: Coverage rate of iodized salt (%) = (Number of households using iodized salt/Total number of surveyed households) × 100%. Iodized salt was defined as household edible salt with an iodine content ≥ 5 mg/kg. The consumption rate of qualified iodized salt was calculated as the proportion of qualified iodized salt among all iodized salt samples, using the formula: Consumption rate of qualified iodized salt (%) = (Number of households using qualified iodized salt/Total number of households using iodized salt) × 100%.

#### 2.3.2. Urinary Iodine

Urinary iodine concentration was determined using the “Determination of Iodine in Urine—Part 1: Arsenic-Cerium Catalytic Spectrophotometry” (WS/T 107.1-2016) [23]. In brief, a 6 mL random urine sample was thawed, thoroughly mixed, and digested with ammonium persulfate to minimize interference from the urinary matrix components. Subsequently, the iodine concentration was calculated using a standard calibration curve established based on the As^3+^–Ce^4+^ catalytic reaction. All participating iodine laboratories were enrolled in both the internal quality control and external quality assurance programs administered by the CDC. Based on the median urinary iodine concentration (MUIC) criteria recommended in the “Guidelines for Monitoring Salt Iodization Programs and Assessing Iodine Nutrition Status in Populations” proposed by the United Nations Children’s Fund (UNICEF) in 2018, a population with an MUIC < 100 μg/L was defined as having iodine deficiency, while a population with an MUIC of 100–299 μg/L was defined as having adequate iodine levels. In this study, participants were categorized into two groups according to their urinary iodine levels: iodine deficiency (MUIC < 100 μg/L) and iodine sufficiency (MUIC ≥ 100 μg/L).

#### 2.3.3. Body Mass Index

Height and weight were measured in strict accordance with the “GBT 26343-2010 Technical Specifications for Student Health Examination” [24], and BMI was calculated as weight/height^2^ (kg/m^2^). According to the Chinese national screening criteria for underweight, overweight, and obesity (WS/T 586-2018 [25], WS/T 456-2014 [26], GB/T 31178-2014 [27]), the participants were subsequently categorized as underweight, normal weight, overweight, or obese.

### 2.4. Quality Control

To ensure data quality, all investigators involved in the project received standardized technical training prior to the survey. During the investigation, unified operational procedures and technical standards were strictly followed to guarantee the authenticity and consistency of questionnaire information. All laboratories participating in sample testing passed the external quality control assessment conducted by the National Iodine Deficiency Disorders Reference Laboratory and possessed the corresponding testing qualifications. Throughout the testing process, internationally certified primary reference materials were used simultaneously for internal quality control. Sample test results were only accepted when the determination values of the reference materials fell within the specified range, thereby ensuring the accuracy and reliability of the testing data.

### 2.5. Statistical Analysis

Statistical analysis was performed using R software (version 4.5.1). Normality was assessed using the Kolmogorov–Smirnov (KS) test. Normally distributed continuous data are presented as mean ± standard deviation ($\bar{x}$ ± *s*). Non-normally distributed continuous data are presented as median (interquartile range) [M (Q1, Q3)]. The Mann–Whitney U test was used for comparisons between two groups. For comparisons among multiple groups, the Kruskal–Wallis H test was employed, followed by the Nemenyi test for pairwise comparisons. Categorical data are presented as rates or proportions, and group differences were compared using the chi-square (*χ*^2^) test. To examine factors associated with iodine nutritional status, logistic regression was performed to identify associated factors. The dependent variable was iodine nutritional status (UIC < 100 μg/L vs. UIC ≥ 100 μg/L), and the independent variables included basic demographic characteristics and personal health behaviors. A stepwise regression method was used to introduce independent variables into the model. *p*-value < 0.05 was considered statistically significant.

## 3. Results

### 3.1. Iodine Content in Salt from the Household Among School-Age Children

In 2022, edible salt samples from 688 households of participants were collected. The median salt iodine concentration was 21.50 mg/kg, with an inter-quartile range (IQR) of 0.00–24.77 mg/kg. Statistically significant differences in salt iodine concentration were observed across different regions (*χ*^2^ = 36.55, *p* < 0.001). Overall, the coverage rate of iodized salt was 64.68%, and the consumption rate of adequately iodized salt among those using iodized salt was 89.89%. Furthermore, significant differences were found in the distribution of household edible salt iodine categories across regions (*χ*^2^ = 82.13, *p* < 0.001). Detailed results are presented in Table 1.

### 3.2. Basic Characteristics and Overall Iodine Nutrition Status of Children and Adolescents Aged 6–17 Years

The median age of the included participants was 11.00 (8.00, 14.00) years. Among them, 378 participants (54.94%) were in the 6–11 years, and 310 participants (45.06%) were in the 12–17 years. There were 356 males (51.74%) and 332 females (48.26%). Participants were distributed across coastal (n = 256, 37.21%), inland (n = 225, 32.70%), and sub-coastal (n = 207, 30.09%) regions. The median urinary iodine concentration (UIC) for all children and adolescents aged 6–17 years was 191.4 μg/L. Based on UIC categories, 105 participants (15.26%) had a UIC < 100 μg/L, while 583 participants (84.74%) had a UIC ≥ 100 μg/L. Details are presented in Table 2 and Table 3.

### 3.3. Distribution of Urinary Iodine Levels Among Children and Adolescents Aged 6–17 Years by Different Demographic Characteristics

Differences in urinary iodine levels were observed among children and adolescents aged 6–17 years across various demographic characteristics. Age-group comparisons revealed that the median urinary iodine concentration (UIC) in the 12–17 years group was 203.4 (132.4, 296.7) μg/L, which was significantly higher than the 181.8 (122.9, 262.3) μg/L observed in the 6–11 years group (*Z* = −2.18, *p* = 0.029). Regional comparisons indicated that the median UIC was highest among children and adolescents in inland areas at 219.7 (143.8, 332.6) μg/L, followed by those in sub-coastal areas at 195.1 (135.8, 271.6) μg/L, and lowest in coastal areas at 160.0 (112.7, 235.2) μg/L, with statistically significant differences among groups (*χ*^2^ = 25.73, *p* < 0.001). The median UIC for individuals from households using iodized salt was 209.6 (136.4, 303.2) μg/L, significantly higher than the 161.6 (110.8, 235.5) μg/L for those from households not using iodized salt (*Z* = −4.47, *p* < 0.001). No statistically significant differences in UIC were found between different genders (*p* = 0.789) or among different BMI-for-age category (*p* = 0.408). Details are presented in Table 2 and Table 3.

### 3.4. Distribution of Urinary Iodine Levels Among Individuals with Different Health Statuses and Lifestyles

No statistically significant differences in the median urinary iodine concentration were found in relation to dietary structure (*p* = 0.508), taste preference (*p* = 0.659), frequency of dining out per week (*p* = 0.974), sedentary behavior (*p* = 0.729), presence of sleep problems (*p* = 0.353), sleep insufficiency (*p* = 0.722), or engagement in moderate-to-vigorous physical activity (MVPA) (*p* = 0.065).

In the comparison of the distribution of urinary iodine levels <100 μg/L versus ≥100 μg/L, only sleep insufficiency showed a statistically significant difference (*χ*^2^ = 3.96, *p* = 0.047). No significant differences were observed for the other health status and lifestyle factors. Details are presented in Supplementary Table S2.

### 3.5. Analysis of Factors Associated with Iodine Nutritional Status in Children and Adolescents Aged 6–17 Years Based on Random Single Urinary Iodine Stratification

In this study, the iodine nutritional status of children and adolescents was used as the dependent variable. Candidate variables for the logistic regression analysis included those from the “Basic Information” and “Personal Health and Behavior” sections; the results of the univariate analysis are presented in Supplementary Table S3. The multivariate logistic regression analysis revealed that age, geographic region, and household consumption of iodized salt were significantly associated with adequate iodine status in children and adolescents. For each one-year increase in age, the likelihood of having adequate iodine status increased (*OR* = 1.10, 95% *CI:* 1.02–1.18, *p* = 0.011). Using coastal areas as the reference, children and adolescents living in inland areas had a higher likelihood of adequate iodine status (*OR* = 1.89, 95% *CI*: 1.03–3.46, *p* = 0.041). Compared with those from households consuming non-iodized salt, individuals from households consuming iodized salt were more likely to have adequate iodine status (*OR* = 1.74, 95% *CI*: 1.09–2.79, *p* = 0.021). The detailed results are shown in Table 4.

## 4. Discussion

Based on data from the 2022 Zhejiang Nutrition and Health Survey, this study found that the median urinary iodine concentration (UIC) among children and adolescents aged 6–17 years in Zhejiang Province was 191.4 μg/L. According to international criteria for assessing iodine nutrition at the population level, this value indicates adequate iodine nutrition [28]. However, a certain proportion of children still exhibited iodine deficiency. Region, age, and household use of iodized salt were associated with the iodine nutritional status of children and adolescents. Notably, children and adolescents in inland areas had relatively higher urinary iodine levels. These findings suggest that, ten years after the adjustment of the national salt iodization policy, the overall iodine nutrition of children and adolescents in Zhejiang Province has reached the optimal level recommended by the World Health Organization [29]. Nevertheless, disparities in iodine intake protection persist across different regions and households. Although the universal salt iodization policy has significantly improved the iodine nutritional status of children and adolescents in China, international experience suggests that iodine deficiency remains a public health concern in certain regions and among specific pediatric subpopulations. A study by Andersson et al., which assessed global iodine nutrition based on median urinary iodine concentrations in school-age children, reported that the proportion of school-age children with low iodine intake globally declined from 36.5% in 2003 to 29.8% in 2011. Nevertheless, approximately 241 million school-age children still had insufficient iodine intake in 2011. Notably, around 74% of children classified as having low iodine intake resided in countries deemed “iodine-sufficient” at the national level, indicating that adequate overall iodine nutrition status does not completely rule out the possibility of insufficient iodine intake among individual children or subgroups [9]. This finding is consistent with the present study, which demonstrated adequate overall iodine nutrition in the study population yet identified a certain proportion of individuals with low urinary iodine levels, further underscoring the public health significance of analyzing the prevalence of low urinary iodine in addition to reporting the median value. Similarly, a study of Spanish children revealed that even when the national median urinary iodine concentration was within the adequate range, approximately 17% of children had urinary iodine levels below 100 μg/L, further suggesting that “overall adequacy” does not necessarily imply that all children are free from the risk of iodine deficiency [11]. A national survey of school-age children in the Philippines indicated that pediatric iodine nutrition could exhibit overall adequacy alongside marked regional disparities, with some areas experiencing iodine deficiency and others showing above-adequate or excessive iodine intake. This pattern aligns with the regional variations in urinary iodine levels and household iodized salt coverage observed in the present study, suggesting that stratified analyses by region may help identify areas requiring focused attention [12]. Furthermore, a study on school-age children in Guinea-Bissau suggested that urinary iodine primarily reflects recent iodine intake, and that pediatric iodine nutritional status should be interpreted in conjunction with household salt iodine content, goiter prevalence, and other dietary and environmental factors. This perspective supports the necessity of incorporating both urinary iodine levels and household salt iodine content in the present analysis [13]. Therefore, this study not only supplements the iodine nutritional surveillance data for children and adolescents at the provincial level in Zhejiang but also demonstrates that, against a backdrop of overall adequate iodine nutrition, it remains necessary to simultaneously monitor the proportion of individuals with low urinary iodine levels, regional disparities, and household iodized salt coverage. This approach provides a basis for identifying key surveillance areas and formulating more targeted intervention measures.

This study found that urinary iodine levels in children and adolescents in coastal areas were lower than those in inland areas, and the median household edible salt iodine content, iodized salt coverage rate, and consumption rate of qualified iodized salt were also lower in coastal areas compared to inland areas. This disparity may be primarily associated with insufficient coverage of qualified iodized salt in households, rather than being solely determined by geographical location, suggesting that coastal areas are not inherently advantageous regions for iodine nutrition in children and adolescents. This finding is consistent with the results reported by Zhao Xuefei et al. [30] in a study conducted in the coastal area of Ningbo: while the overall iodine nutrition status of local children and adolescents was adequate, the use of non-iodized salt persisted, indicating a need to continuously reinforce the universal salt iodization strategy. Although seafood can contribute to dietary iodine intake, the amount consumed varies significantly among individuals and cannot reliably replace qualified iodized salt as a stable and consistent source of dietary iodine [31]. Coastal residents may also hold the misconception that consuming seafood can completely substitute for iodized salt, or they may have greater access to non-iodized sea salt. Therefore, the coastal-inland disparity observed in this study reflects, to a greater extent, differences in household behaviors regarding the selection and use of iodized salt, rather than mere differences in food resources or the natural environment. Furthermore, this study found that household consumption of iodized salt was associated with the iodine nutritional status of children and adolescents. Both univariate and multivariate analyses indicated that children and adolescents who consumed iodized salt were more likely to have adequate iodine status. This suggests that in the context of increasingly diversified dietary sources for residents, household iodized salt remains an important source of stable iodine intake among children and adolescents. This finding is consistent with the conclusion of Chen Fang et al. [8], who reported that even in areas with high iodized salt coverage, non-compliant iodized salt and improper household storage and usage may be associated with reduced effectiveness of iodine supplementation. Additionally, research by Lang Rui [32] showed that iodized salt coverage and the consumption rate of qualified iodized salt have declined compared to the past, indicating that during the scientific and precise iodine supplementation phase, continuous attention to household iodine supplementation behaviors is necessary. Therefore, compared to factors that are more difficult to change, such as age and region, improving the coverage and standardized usage rate of qualified household iodized salt possesses greater potential for intervention and public health practice value.

Additionally, age was associated with iodine nutritional status, with younger children potentially being a subgroup requiring greater attention. As age increases, food intake and dietary diversity may expand, leading to a corresponding increase in iodine intake pathways. In contrast, the diets of younger children are more dependent on family provision, making them more susceptible to household salt choices and dietary patterns. These findings are consistent with those reported by Li Xueqing et al. [33], indicating that thyroid-related outcomes in children and adolescents exhibit age heterogeneity, which suggests that more targeted monitoring and intervention strategies should be adopted for different age groups.

This study suggests that, in the context of an overall adequate iodine nutrition level, the focus of iodine deficiency disorder prevention and control should shift towards key regions and household behavior management. Particularly, ten years after the adjustment of the national salt iodization policy, greater attention should be paid to the decline in household coverage of qualified iodized salt in coastal areas and its potential impact on iodine intake among children and adolescents. Health education targeting parents and guardians should be strengthened to correct misconceptions such as “seafood can replace iodized salt”. Concurrently, emphasis should be placed on the proper selection, storage, and consistent use of qualified iodized salt to enhance the actual iodine supplementation effect at the household level.

This study has several limitations. First, its cross-sectional design precludes causal inference; thus, the observed associations should not be interpreted as causal relationships. Second, although spot urine samples are appropriate for assessing population-level iodine nutrition status based on median urinary iodine concentration (UIC), a single spot urine sample is insufficient to accurately reflect an individual’s long-term stable iodine intake, as UIC may vary with recent dietary intake, hydration status, and time of specimen collection. Third, the primary analysis included only participants with complete data on urinary iodine, household salt iodine, geographic classification, and key questionnaire variables. While a comparison between participants with available UIC data and the final analytical sample suggested that the overall assessment of iodine status was not substantially altered, potential selection bias cannot be entirely ruled out. Fourth, this study did not collect information regarding household knowledge, purchasing preferences, or storage and usage practices related to iodized salt. Consequently, the interpretation of the underlying mechanisms contributing to regional differences requires further validation through subsequent investigations.

## 5. Conclusions

Overall, the iodine nutritional status of children and adolescents aged 6–17 years in Zhejiang Province was generally adequate at the population level; however, iodine deficiency was still observed in some individuals. Age, geographic region, and household consumption of iodized salt were associated with the achievement of adequate iodine nutrition. Specifically, children and adolescents residing in coastal areas exhibited relatively lower iodine nutritional levels. In the future, targeted monitoring should be intensified in coastal regions and among younger children. Furthermore, region-specific health education and guidance on the use of iodized salt should be implemented, taking into account the local iodine nutritional status and household salt consumption patterns.

## Figures and Tables

**Table 1:** Household Salt Iodine Status among Children and Adolescents Aged 6–17 Years in Zhejiang Province, 2022.

Variables	Coastal Area	Inland Area	Sub-Coastal Area	Total
Salt Iodine Concentration (mg/kg) [M **(Q_1_, Q_3_)**]	0.00 (0.00, 24.00)	22.89 (18.95, 25.25)	22.04 (0.00, 24.68)	
Statistic	*χ*^2^ = 36.55 ^#^			
*p*	<0.001			
Classification of Iodized Salt				
Unqualified Iodized Salt	22 (48.89)	8 (17.78)	15 (33.33)	45
Non-iodized salt	138 (56.79)	43 (17.70)	62 (25.51)	243
Qualified iodized salt	96 (24.00)	174 (43.50)	130 (32.50)	400
Statistic	*χ*^2^ = 82.13			
*p*	<0.001			
Coverage rate of iodized salt (%)	46.09	80.89	70.05	64.68
Qualified iodized salt consumption rate (%)	81.36	95.6	89.66	89.89
Total, n (%)	256 (37.21)	225 (32.70)	207 (30.09)	688 (100.00)

^#^: Kruskal–Wallis test, *χ*^2^: Chi-square test, M: Median, Q_1_: 1st Quartile, Q_3_: 3st Quartile.

**Table 2:** Urinary Iodine Concentration by Demographic Characteristics among Children and Adolescents Aged 6–17 Years in Zhejiang Province.

Variable	Overall, n (%)	UIC (μg/L), M (Q_1_, Q_3_)	Statistic	*p*
Age group, n (%)			*Z* = −1.92	0.055
6–11	378 (54.94)	181.8 (122.9, 262.3)		
12–17	310 (45.06)	203.4 (132.4, 296.7)		
Gender, n (%)			*Z* = −0.27	0.789
Boy	356 (51.74)	182.9 (130.4, 269.6)		
Girls	332 (48.26)	201.6 (121.2, 282.7)		
Region, n (%)			*χ*^2^ = 25.73 ^#^	<0.001
Coastal area	256 (37.21)	160.0 (112.7, 235.2)		
Inland area	225 (32.70)	219.7 (143.8, 332.6)		
Sub-coastal area	207 (30.09)	195.1 (135.8, 271.6)		
BMI-for-age category, n (%)			*χ*^2^ = 2.90 ^#^	0.408
Underweight	134 (19.48)	179.6 (118.0, 259.4)		
Normal	408 (59.30)	192.3 (128.0, 276.3)		
Overweight	89 (12.94)	202.2 (136.0, 276.0)		
Obese	57 (8.28)	169.8 (126.9, 237.3)		
Iodized salt, n (%)			*Z* = −4.47	<0.001
Non-iodized salt	243 (35.32)	161.6 (110.8, 235.5)		
Iodized salt	445 (64.68)	209.6 (136.4, 303.2)		

UIC: urinary iodine concentration; ^#^: Kruskal–Wallis test, *χ*^2^: Chi-square test, Z: Mann–Whitney test; M: Median, Q_1_: 1st Quartile, Q_3_: 3st Quartile.

**Table 3:** Iodine Status Distribution by Demographic Characteristics among Children and Adolescents Aged 6–17 Years in Zhejiang Province.

Variable	Overall, n (%) or M (Q1, Q3)	UIC < 100 μg/L, n (%) or M (Q1, Q3)	UIC ≥ 100 μg/L, n (%) or M (Q1, Q3)	Statistic	*p*
Age, M (Q1, Q3)	11.00 (8.00, 14.00)	10.00 (8.00, 13.00)	11.00 (8.00, 14.00)	*Z* = −2.18	0.029
Age group, n (%)				*χ*^2^ = 2.43	0.119
6–11	378 (54.94)	65 (61.90)	313 (53.69)		
12–17	310 (45.06)	40 (38.10)	270 (46.31)		
Gender, n (%)				*χ*^2^ = 2.42	0.120
Boy	356 (51.74)	47 (44.76)	309 (53.00)		
Girls	332 (48.26)	58 (55.24)	274 (47.00)		
Region, n (%)				*χ*^2^ = 6.25	0.044
Coastal area	256 (37.21)	50 (47.62)	206 (35.33)		
Inland area	225 (32.70)	26 (24.76)	199 (34.13)		
Sub-coastal area	207 (30.09)	29 (27.62)	178 (30.53)		
BMI, M (Q1, Q3)	17.55 (15.22, 20.15)	16.66 (14.88, 19.51)	17.75 (15.31, 20.37)	*Z* = −2.21	0.027
BMI-for-age category, n (%)				*χ*^2^ = 5.80	0.122
Underweight	134 (19.48)	23 (21.90)	111 (19.04)		
Normal	408 (59.30)	69 (65.71)	339 (58.15)		
Overweight	89 (12.94)	8 (7.62)	81 (13.89)		
Obese	57 (8.28)	5 (4.76)	52 (8.92)		
Iodized salt, n (%)				*χ*^2^ = 6.98	0.008
Non-iodized salt	243 (35.32)	49 (46.67)	194 (33.28)		
Iodized salt	445 (64.68)	56 (53.33)	389 (66.72)		

For continuous variables, values are presented as M (Q1, Q3); for categorical variables, values are presented as n (%). UIC: urinary iodine concentration; M: median; Q1: first quartile; Q3: third quartile. *χ*^2^: chi-square test; Z: Mann–Whitney U test.

**Table 4:** Multivariate Logistic Regression Analysis of Factors Associated with Iodine Nutritional Status among Children and Adolescents Aged 6–17 Years in Zhejiang Province.

Variables	*β*	*S.E.*	*Z*	*p*	*OR* (95%*CI*)
Age	0.09	0.04	2.54	0.011	1.10 (1.02~1.18)
Region					
Coastal area					1.00 (Reference)
Sub-coastal area	0.13	0.28	0.44	0.659	1.13 (0.65~1.98)
Inland area	0.63	0.31	2.05	0.041	1.89 (1.03~3.46)
Iodized Salt					
Non-iodized salt					1.00 (Reference)
Iodized salt	0.56	0.24	2.32	0.021	1.74 (1.09~2.79)

## Data Availability

The data presented in this study are available on request from the corresponding author. The data are not publicly available due to them involving personal information.

## Supplementary Materials

The following supporting information can be downloaded at: https://www.mdpi.com/article/10.3390/nu18101634/s1, Table S1: Definitions of personal health and behavioral variables included in the analysis; Table S2: Comparison of Urinary Iodine Levels among Children and Adolescents Aged 6–17 Years in Zhejiang Province by Health Status and Lifestyle; Table S3. Univariate Logistic Regression Analysis of Factors Associated with Iodine Nutritional Status among Children and Adolescents Aged 6–17 Years in Zhejiang Province.
