# Supplementary material for: Iodine Nutritional Status and Its Associated Factors Among Children and Adolescents in Zhejiang Province Ten Years After the Downward Adjustment of the National Salt Iodization Policy
Source: Nutrients. 2026 May 21;18(10):1634. doi: 10.3390/nu18101634 (PMC13209854; doi:10.3390/nu18101634)
Supplement: Supplementary file 1 [file nutrients-18-01634-s001.zip › nutrients-4298787-supplementary.pdf]

nutrients

MDPI

Supplementary material

Table S1. Definitions of personal health and behavioral variables included in the analysis.

Variable	Categories used in the analysis	Definition and classification
Dietary characteristics	Predominantly vegetarian; Predominantly meat-based; Balanced meat and vegetable intake	Dietary characteristics were assessed using a standardized personal health and behavior questionnaire. Participants were classified based on predefined, self-reported response options: predominantly vegetarian, predominantly meat-based, or balanced meat and vegetable intake. No additional quantitative threshold was applied to these categorical responses.
Dietary taste preference	Preference for salty foods; Moderate salt preference; Preference for bland foods	Dietary taste preference was assessed using the standardized questionnaire and classified according to self-reported response options: preference for salty foods, moderate salt preference, or preference for bland foods.
Average frequency of dining out per week	≤2 times; 3–6 times; 7–10 times; >10 times	Dining-out frequency was defined as the self-reported average weekly frequency of dining out, excluding breakfast. Participants were classified according to the predefined questionnaire categories: ≤2 times, 3–6 times, 7–10 times, or >10 times per week.
Sedentary behavior	False; True	Sedentary behavior was derived from the Physical Activity Questionnaire for Children and Adolescents (6–17 years). For children and adolescents, total sedentary time was calculated by summing the reported time spent in sedentary activities on both school days and rest days. These activities included non-screen-based study, screen-based study, watching television or videos, using mobile phones, playing video games, using computers or tablets, and reading paper books, magazines, or newspapers. Sleep time was not included. In this study, the larger value of total sedentary time between school days and rest days was used. Sedentary behavior was defined as total sedentary time ≥1 hour per day.

https://doi.org/10.3390/nu18101634

Sleep problem	False; True	Sleep problems were assessed using the Physical Activity Questionnaire for Children and Adolescents (6–17 years). Participants were classified as having a sleep problem if they reported at least one of the following symptoms occurring on at least 3 days per week during the past 30 days: snoring, choking or suffocation during sleep, difficulty falling asleep, waking up at least twice during the night with difficulty returning to sleep, use of sleeping medication at least once, or early awakening with difficulty returning to sleep.
Insufficient sleep	False; True	Sleep duration was calculated based on self-reported daytime and nighttime sleep duration on school days and rest days. For children and adolescents, the shorter duration between school days and rest days was used as the final sleep duration. Insufficient sleep was defined as sleep duration <9 hours per day for participants aged 6–13 years and <8 hours per day for participants aged 14–17 years.
Moderate-to-vigorous physical activity	False; True	Moderate-to-vigorous physical activity (MVPA) was derived from the Physical Activity Questionnaire for Children and Adolescents (6–17 years). Participants were classified as engaging in MVPA if they reported having any moderate- or vigorous-intensity activity in their daily life during the current semester. Moderate- or vigorous-intensity activities were described in the questionnaire as activities that could cause increased breathing, sweating, or increased heart rate, such as running, cycling, swimming, playing, housework, or physical exercise.

Table S2. Comparison of Urinary Iodine Levels among Children and Adolescents Aged 6–17 Years in Zhejiang Province by Health Status and Lifestyle.

Variables	n（%）	Urinary iodine concentration (μg/L), M (Q_1, Q_3)	Statistic	*P*	Urinary iodine concentration distribution (n,%)		Statistic	*P*
					<100 μg/L	≥100 μg/L		
Dietary Characteristics, n(%)			χ^2=1.35[#]	0.508			χ^2=0.08	0.961
Predominantly vegetarian	17 (2.64)	225.0 (145.6, 271.8)			3 (2.86)	14 (2.40)		
Predominantly meat-based	66 (10.25)	174.9 (125.0, 243.4)			10 (9.52)	56 (9.61)		
Balanced meat and vegetable intake	561 (87.11)	190.9 (128.0, 272.4)			85 (80.95)	476 (81.65)		
Dietary Taste Preference, n(%)			χ^2=0.83[#]	0.659			χ^2=0.57	0.754
Preference for salty foods	48 (7.45)	175.7 (122.8, 254.3)			9 (8.57)	39 (6.69)		
Moderate salt preference	501 (77.80)	192.1 (129.1, 271.4)			74 (70.48)	427 (73.24)		
Preference for bland foods	95 (14.75)	181.9 (121.1, 252.3)			15 (14.29)	80 (13.72)		
Average Frequency of Dining Out per Week, n(%)			χ^2=0.22[#]	0.974			-	0.665
<=2times	517 (80.16)	191.4 (123.6, 276.0)			84 (80.00)	433 (74.27)		
3-6times	85 (13.18)	196.9 (143.7, 254.9)			11 (10.48)	74 (12.69)		
7-10times	26 (4.03)	179.1 (144.9, 257.9)			2 (1.90)	24 (4.12)		

＞10times	17 (2.64)	176.3 (136.9, 232.4)			2 (1.90)	15 (2.57)		
Sedentary, n(%)			*Z*=-0.35	0.729			χ^2=0.07	0.785
FALSE	14 (2.03)	188.4 (151.4, 227.8)			3 (2.86)	11 (1.89)		
TRUE	674 (97.97)	191.4 (127.6, 274.2)			102 (97.14)	572 (98.11)		
Sleep Problem, n(%)			*Z*=-0.93	0.353			χ^2=0.73	0.393
FALSE	647 (94.18)	192.0 (128.0, 272.5)			97 (92.38)	550 (94.34)		
TRUE	40 (5.82)	171.7 (117.8, 283.8)			8 (7.62)	32 (5.49)		
Insufficient sleep, n(%)			*Z*=-0.36	0.722			χ^2=3.96	0.047
FALSE	432 (62.79)	191.3 (125.5, 274.8)			75 (71.43)	357 (61.23)		
TRUE	256 (37.21)	191.7 (129.6, 270.7)			30 (28.57)	226 (38.77)		
Moderate Vigorous Activity, n(%)			*Z*=-1.84	0.065			χ^2=1.22	0.269
FALSE	208 (30.28)	203.8 (138.8, 284.4)			27 (25.71)	181 (31.05)		
TRUE	479 (69.72)	183.6 (123.0, 270.2)			78 (74.29)	401 (68.78)		
Total	688	191.4(127.9, 272.7)			105	583		

#: Kruskal-Wallis test, χ^2: Chi-square test,Z: Mann-Whitney test
M: Median, Q_1: 1st Quartile, Q_3: 3st Quartile

Table S3. Univariate Logistic Regression Analysis of Factors Associated with Iodine Nutritional Status among Children and Adolescents Aged 6–17 Years in Zhejiang Province.

Variables	*β*	*S.E*	*Z*	*P*	*OR* (95%*CI*)
Age	0.07	0.03	2.13	0.033	1.07 (1.01 ~ 1.14)
Region					
Coastal area					1.00 (Reference)
Sub-coastal area	0.40	0.25	1.56	0.118	1.49 (0.90 ~ 2.46)
Inland area	0.62	0.26	2.37	0.018	1.86 (1.11 ~ 3.10)
Gender					
Male					1.00 (Reference)
Female	-0.33	0.21	-1.55	0.121	0.72 (0.47 ~ 1.09)
Sleep Problem					
FALSE					1.00 (Reference)
TRUE	-0.35	0.41	-0.85	0.395	0.71 (0.32 ~ 1.58)
Iodized Salt					
Non-iodized salt					1.00 (Reference)
Iodized salt	0.56	0.21	2.62	0.009	1.75 (1.15 ~ 2.67)
Dietary Characteristics, n(%)					
Predominantly vegetarian					1.00 (Reference)
Predominantly meat-based	0.18	0.72	0.25	0.801	1.20 (0.29 ~ 4.95)
Balanced meat and vegetable intake	0.18	0.65	0.28	0.778	1.20 (0.34 ~ 4.27)
Dietary Taste Preference, n(%)					
Preference for salty foods					1.00 (Reference)
Moderate salt preference	0.29	0.39	0.73	0.464	1.33 (0.62 ~ 2.86)
Preference for bland foods	0.21	0.46	0.45	0.655	1.23 (0.50 ~ 3.06)
Average Frequency of Dining Out per Week, n(%)					
<=2times					1.00 (Reference)

3-6times	0.27	0.34	0.77	0.440	1.31 (0.66 ~ 2.56)
7-10times	0.84	0.75	1.13	0.257	2.33 (0.54 ~ 10.04)
＞10times	0.37	0.76	0.49	0.623	1.45 (0.33 ~ 6.48)
Sleep Deprivation					
FALSE					1.00 (Reference)
TRUE	0.46	0.23	1.98	0.048	1.58 (1.01 ~ 2.49)
Moderate Vigorous Activity					
FALSE					1.00 (Reference)
TRUE	-0.27	0.24	-1.10	0.270	0.77 (0.48 ~ 1.23)
Bmi Group					
Normal					1.00 (Reference)
Obese	0.75	0.49	1.54	0.123	2.12 (0.82 ~ 5.49)
Overweight	0.72	0.39	1.84	0.066	2.06 (0.95 ~ 4.46)
Underweight	-0.02	0.26	-0.07	0.946	0.98 (0.58 ~ 1.65)
